# The Value of Reducing Inconclusive and False-Positive Newborn Screening Results for Congenital Hypothyroidism, Congenital Adrenal Hyperplasia and Maple Syrup Urine Disease in The Netherlands

**DOI:** 10.3390/ijns10040070

**Published:** 2024-10-08

**Authors:** Rosalie C. Martens, Anita Boelen, Michèle H. van der Kemp, Annet M. Bosch, Eveline M. Berghout, Gert Weijman, Nitash Zwaveling-Soonawala, Rendelien K. Verschoof-Puite, Robert de Jonge, Sabine E. Hannema, Judith E. Bosmans, Annemieke C. Heijboer

**Affiliations:** 1 Endocrine Laboratory, Department of Laboratory Medicine, Amsterdam University Medical Centers, Location University of Amsterdam, 1105 AZ Amsterdam, The Netherlands; 2 Endocrine Laboratory, Department of Laboratory Medicine, Amsterdam University Medical Centers, Location Vrije Universiteit Amsterdam, 1081 HV Amsterdam, The Netherlands; 3 Amsterdam Gastroenterology, Endocrinology & Metabolism, 1105 AZ Amsterdam, The Netherlands; 4 Amsterdam Reproduction and Development Research Institute, 1105 AZ Amsterdam, The Netherlands; 5 Value-Based Healthcare Strategy & Tactics, VDKMP, 1017 XP Amsterdam, The Netherlands; 6 Department of Pediatrics, Division of Metabolic Diseases, Emma Children’s Hospital, Amsterdam University Medical Centers, Location University of Amsterdam, 1105 AZ Amsterdam, The Netherlands; 7 Department of Pediatrics, Deventer Ziekenhuis, 7417 SE Deventer, The Netherlands; 8 Department for Vaccine Supply and Prevention Programs, RIVM Dutch National Institute for Public Health and the Environment, 3720 BA Bilthoven, The Netherlands; 9 Department of Pediatric Endocrinology, Emma Children’s Hospital, Amsterdam University Medical Centers, Location University of Amsterdam, 1105 AZ Amsterdam, The Netherlands; 10 Department of Laboratory Medicine, Amsterdam University Medical Centers, Location University of Amsterdam, 1105 AZ Amsterdam and Location Vrije Universiteit Amsterdam, 1081 HV Amsterdam, The Netherlands; 11 Department of Pediatrics, Amsterdam University Medical Centers, Location Vrije Universiteit Amsterdam, 1081 HV Amsterdam, The Netherlands; 12 Department of Health Sciences, Faculty of Science, Amsterdam Public Health Research Institute, Vrije Universiteit Amsterdam, 1081 HV Amsterdam, The Netherlands

**Keywords:** congenital adrenal hyperplasia, congenital hypothyroidism, false-positive result, maple syrup urine disease, newborn screening, financial benefits

## Abstract

Inconclusive and false-positive newborn screening (NBS) results can cause parental stress and increase healthcare expenditures. These results can be reduced by improving NBS algorithms. This was recently done for Congenital Hypothyroidism (CH), Congenital Adrenal Hyperplasia (CAH) and Maple Syrup Urine Disease (MSUD) in the Dutch NBS program. The current study estimates the financial consequences of these improved algorithms related to the reduction in inconclusive results and false-positives. For each improved algorithm, the care pathway of an inconclusive/false-positive result was analyzed. The costs associated with the improvements, based on the change in inconclusive results/false-positives, were assessed to estimate the cost reduction per year. The improvements resulted in a reduction of inconclusive results and/or false-positives, without increasing false-negatives. For CH, false positives decreased by 26 per year with a related cost reduction of EUR 31,156. For CAH, 95 second heel punctures and seven false-positives per year were avoided, leading to a related cost reduction of EUR 7340. For MSUD, five false-positives per year were avoided with a related cost reduction of EUR 11,336. The improved screening algorithms led to a cost reduction of EUR 49,832 annually. Together with the known negative psychosocial effects associated with an inconclusive or false-positive NBS result, these results highlight the importance of improving NBS algorithms.

## 1. Introduction

Currently, the Dutch Newborn Screening (NBS) program screens for 27 congenital disorders [1]. Yearly, approximately 170,000 children are screened in the Netherlands. The costs per screened child were EUR 146 in 2022 [2]. As is inherent in any screening program, results of screening may be inconclusive or false positive. An inconclusive result requires a second heel puncture that can lead to either a negative screening or a referral. A false-positive result means that the initial screening result is positive, but after referral to the pediatrician and extra diagnostic tests, the screening result cannot be confirmed and the neonate does not have the disease. Both a second heel puncture and a false-positive NBS result have an impact on the child, its parents and the healthcare system. According to a recently published Dutch study, parents of a child with an inconclusive or false-positive NBS result experience more negative emotions in the first 5 weeks after the NBS result than parents who received a negative (normal) NBS result [3]. Parents of a child with a false-positive NBS result still experience more negative emotions after four months compared to parents of a child with a negative NBS result. Several other studies observed a similar impact on parental stress in the first year after the initial NBS result [4,5,6,7,8]. Parents were particularly worried about symptoms related to the disease for which they were referred on the basis of the NBS result [5].

Additionally, false-positive and inconclusive NBS results were associated with higher self-reported healthcare utilization in the Netherlands, not related to the initial visits after a positive result [3]. Children with a false-positive NBS result visited the pediatrician more often in the first four months after the NBS result and had more hospital day treatment admissions and other hospital admissions in the first five weeks after the NBS result. Children with an inconclusive result had more emergency department visits in the first five weeks after the NBS result. This was also found in studies performed outside the Netherlands [4,9,10]. Reasons given were that healthcare professionals were unfamiliar with the disease for which the child had an inconclusive or false-positive result and therefore the child was referred more easily, and that parents felt their child was more fragile. However, other studies did not observe an increase in healthcare utilization [11,12].

An inconclusive or false-positive result also leads to preventable healthcare expenditures and workload for healthcare professionals related to the follow-up in the case of an inconclusive or false-positive result. In the case of an inconclusive result, a second heel puncture needs to be performed, while a false-positive result leads to a referral to the pediatrician, together with additional diagnostic tests to confirm the positive NBS result. This is not only burdensome for the parents and children but also increases healthcare expenditures, which could be at least partly prevented with a better screening algorithm.

Thus, there are several psychosocial and economic reasons that make it important to reduce the number of inconclusive and false-positive results in neonatal screening. One way to do this is by improving screening algorithms. In 2020 and 2021, Stroek et al. evaluated the screening algorithms for Congenital Hypothyroidism (CH), Congenital Adrenal Hyperplasia (CAH) and Maple Syrup Urine Disease (MSUD) and recommended improvements to reduce inconclusive and/or false-positive results, without increasing the number of false negatives [13,14,15]. Based on these evaluations, the improved screening algorithms were all implemented in the Dutch NBS in 2021, the new algorithms can be found in the Supplementary Materials. The aim of this study was to estimate the value of the reduction in inconclusive and/or false-positive results due to the recent improvements in the Dutch NBS algorithms for CH, CAH and MSUD. We express the value of these improvements as financial benefits.

## 2. Methods

### 2.1. Case 1—Congenital Hypothyroidism

In 2021, Stroek et al. published the evaluation of the NBS CH algorithm, which aimed to lower the number of false-positive results due to partial thyroxin binding globulin (TBG) deficiency [13]. A modification in the cut-off value for TBG from ≤40 nmol/L blood to ≤105 nmol/L blood would reduce the number of false-positive results without increasing the number of false-negative results. This modification does not affect the number of inconclusive results as it diminishes the number of referrals due to a low T4 concentration (<−3 SD) due to (partial) TBG deficiency. Therefore, this case focuses only on the false-positives. The newly implemented cut-off value of ≤105 nmol/L blood was based upon expert opinion combined with the two studies on evaluating and improving CH NBS by Stroek et al. [13,16]. The adjusted NBS algorithm was implemented in the NBS from March 2021 in the Netherlands. The number of false-positives before and after the improvement was based upon unpublished data of Amsterdam UMC, based on the articles by Stroek et al. [13,16].

### 2.2. Case 2—Congenital Adrenal Hyperplasia

In 2021, the evaluation of the CAH NBS algorithm was published by Stroek et al., which aimed to reduce the number of second heel punctures (due to an inconclusive result) and false-positive results [14]. Prior to the evaluation, the CAH NBS algorithm was only based on the analysis of 17-hydroxyprogesterone (17-OHP) concentration in dried blood spots (DBS) and a second heel puncture had to be performed in the case of an inconclusive result. The evaluation of Stroek et al. showed that adding a second-tier test for 21-deoxycortisol (21-DOCL, a highly specific marker for CAH) in the same DBS in the case of an inconclusive 17-OHP result prevented the second heel punctures and resulted in a reduction of false-positive results. Therefore, this case focuses on the reduction of inconclusive results as well as on the reduction of false-positives. This adjustment was implemented in the Dutch NBS program in October 2021. The number of inconclusive and false-positive results of the old and new screening algorithm was based upon the evaluation study published by Stroek et al. [14].

### 2.3. Case 3—Maple Syrup Urine Disease

In 2020, Stroek et al. published an evaluation of the NBS algorithm for MSUD, aiming to reduce only the number of false-positives asthe MSUD screening algorithm does not include an inconclusive category. The evaluation suggested adding a ratio of Leucine (Xle)/Phenylalanine (Phe) ≥ 5.0 to the already available markers Xle and Valine (Val) (≥340 µmol/L) in the screening algorithm [15]. Before the evaluation of Stroek et al., the cut-off values of Xle and Val had already been adapted from ≥400 µmol/L to ≥340 µmol/L in 2019 with the use of a new screening kit. The improvement of the added ratio of Xle/Phe, followed by the evaluation of Stroek et al., was approved by the National Institute for Public Health and the Environment (RIVM) and implemented in the Dutch NBS program in October 2021. The number of false positives was based upon unpublished data of the National Institute for Public Health and the Environment (RIVM). They tested both the old algorithm (Xle and Val ≥ 340 µmol/L) and the new algorithm (Xle and Val ≥ 340 µmol/L and ratio Xle/Phe ≥ 5) on a cohort from 2018 to mid-2020.

### 2.4. Analysis

For each improved screening algorithm (CH, CAH and MSUD), we examined whether the improved screening algorithm was associated with fewer inconclusive and false-positive results, and lower or equal costs compared to the previously used screening algorithm. First, the care pathway of an inconclusive and/or false-positive NBS result per case was described in collaboration with a multidisciplinary team. Secondly, the costs of the described care pathways were estimated, and the change in costs due to the reduction in the number of inconclusive and false-positive results based on the improved algorithms was estimated. The types of costs are shown in Table 1 and were derived from the National Institute for Public Health and the Environment (RIVM), the reference prices of the Dutch costing manual for economic evaluations in healthcare and the Dutch DBC (diagnosis treatment combination) tariffs (Table 1) [17,18]. The cost differences per year were calculated by multiplying the reduction in inconclusive/false-positive results during the total follow-up period in the studies by Stroek et al. with the costs associated with an inconclusive/false-positive result and they were thereafter divided by the time period (in years) of the follow-up period. The cost calculation was performed with non-rounded numbers, and after calculation the final result was rounded. Below, the improvement in the algorithm per case is described.

## 3. Results 

### 3.1. Care Pathways

#### 3.1.1. False-Positive NBS Result CH, CAH and MSUD

**Figure 1:**
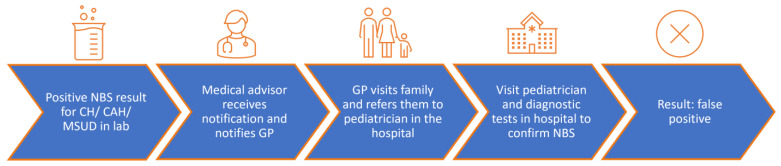
Care pathway for a false-positive NBS result for CH, CAH and MSUD. NBS newborn screening; CH Congenital Hypothyroidism; CAH Congenital Adrenal Hyperplasia; MSUD Maple Syrup Urine Disease; GP General Practitioner.

Figure 1 shows the care pathway of a false-positive NBS result. This care pathway is similar for CH, CAH and MSUD and consists of the following components. First, a medical advisor of the Dutch NBS program receives a notification of the positive screening result from the screening laboratory and notifies the General Practitioner (GP), and for MSUD also the pediatrician for metabolic diseases. The GP contacts the family and refers the infant to a pediatrician in the case of a positive CH result, to a pediatric endocrinologist in the case of a positive CAH result and to a pediatrician for metabolic diseases in the case of a positive MSUD result. The pediatrician evaluates the infant and requests diagnostic (blood) tests to confirm the positive screening result. To confirm the diagnosis of CH, plasma TSH and free T4 are measured [19]. For CAH 17-OHP, androstenedione and 21-DOCL are determined to confirm the diagnosis, and sodium, potassium and plasma renin activity will be analyzed in serum to assess salt-wasting [20]. For MSUD, amino acids will be analyzed in plasma and organic acids in urine, and if necessary, BCKA-dehydrogenase activity measurement and genetic studies will also be performed [21]. Based on the results of these diagnostic (blood) tests, the NBS result can turn out to be false positive or true positive.

#### 3.1.2. Inconclusive NBS Result CAH

**Figure 2:**
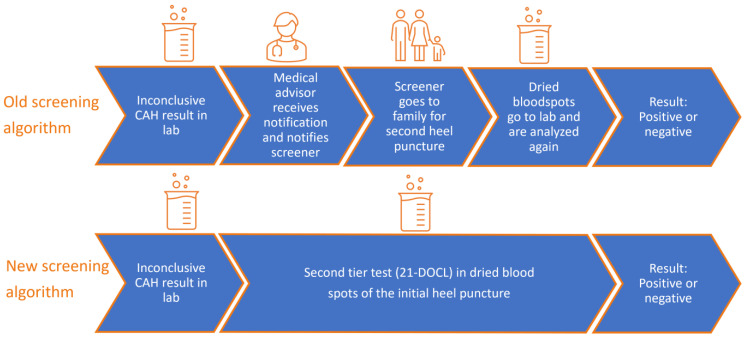
Previous and current care pathways in the case of an inconclusive screening result for CAH. CAH Congenital Adrenal Hyperplasia; 21-DOCL 21-deoxycortisol.

Figure 2 shows the care pathway of an inconclusive result for CAH before and after the improvement by Stroek et al. Before the improvement, in the case of an inconclusive result for CAH, a second heel puncture was performed. The care pathway of a second heel puncture was as follows: the screening laboratory finds an inconclusive result, and the medical advisor of the Dutch NBS programs receives a notification of this result and notifies the screener to perform a second heel puncture. The screener sends the DBS of this second heel puncture to the screening laboratory to analyze 17-OHP again. After the improvement, in the case of an inconclusive result, the laboratory performs the second-tier test (21-DOCL) in the already available DBS from the first heel puncture, which substitutes the second heel puncture.

### 3.2. Case 1—Congenital Hypothyroidism

#### Volumes and Costs of False-Positive NBS Result CH

The adaptation of the cut-off value of TBG in the CH algorithm did not increase the costs of the screening itself but did result in a reduction of 285 false positives (from 2183 to 1898 false positives) over 11 years, which is a reduction of almost 26 false positives per year. The costs of the care pathway related to a false-positive CH screening were a total of EUR 1203 per false-positive result (see Table 2 for specifications). The prevented false positives associated with the improved algorithm therefor resulted in a cost reduction of EUR 31,156 per year. 

### 3.3. Case 2—Congenital Adrenal Hyperplasia

#### 3.3.1. Volumes and Costs of Inconclusive NBS Result CAH

The addition of the second tier (21-DOCL) in the CAH algorithm prevents all second heel punctures, which numbered 286 over three years, 2017–2019 (±95 per year) [14]. Table 3 shows that the costs of a second heel puncture were EUR 272 and the costs of the second-tier test were EUR 290, resulting in a cost increase per inconclusive result of EUR 18. Thus, the total costs associated with an inconclusive CAH result increased by EUR 1664 per year after the improvement in the algorithm.

#### 3.3.2. Volumes and Costs of False-Positive NBS Result CAH

The addition of the second tier to the CAH algorithm prevented 22 false positives (from 33 to 11 false positives) over three years, which is a reduction of approximately 7 false-positives per year [14]. These false-positive results were all obtained after an initial inconclusive result followed by a second heel puncture that was positive. This was prevented by the second tier. Table 4 shows the specification of the costs of the care pathway associated with a false-positive CAH screening, which were in total EUR 1228 per false positive. Thus, a total cost reduction of EUR 9004 per year was achieved after the improvement due to the reduction in the number of false positives.

#### 3.3.3. Total Costs of Inconclusive and False-Positive NBS Result CAH

The total costs of the CAH screening reduced by EUR 7340 per year after implementation of the improved algorithm. This consists of the cost savings due to a reduction in false positives (EUR 9004/year) minus the increase in costs of the second-tier test compared to the second heel puncture in the case of an inconclusive result (EUR 1664/year). 

### 3.4. Case 3—Maple Syrup Urine Disease

#### Volumes and Costs of False-Positive NBS Result MSUD

The addition of the Xle/Phe ratio did not increase the cost of the screening itself and resulted in a reduction of 13 false positives (from 17 to 4 false positives) over 2.5 years (cohort 2018–mid-2020), which is on average 5 false positives per year. The costs of the care pathway associated with a false-positive MSUD result were in total EUR 2180 (Table 5). The reduction in the number of false-positives due to the improved algorithm resulted therefor in a cost reduction of EUR 11,336 per year.

## 4. Discussion

The aim of this study was to evaluate the financial benefits gained by the reduction in the number of inconclusive and false-positive results after improvements in the NBS algorithms for CH, CAH and MSUD. The improved algorithm for CH showed a reduction of 26 false positives per year and a related cost reduction of EUR 31,116 per year. For CAH, the reduction in second heel punctures was 95 per year and in false-positives seven per year, resulting in a total cost reduction of EUR 7340 per year. The improved algorithm for MSUD showed a reduction of five false-positives per year and a related cost reduction of EUR 11,336 per year. 

These results show that the improvement in the CH algorithm had the largest impact on costs. This can be explained by the relatively high prevalence of CH and the relatively high number of false positives in the previous CH algorithm. This relatively high number of false positives is due to the fact that the Dutch CH NBS algorithm uses T4 as the primary marker, which is less specific than TSH. However, this approach leads to the detection of not only primary/thyroidal CH but also central CH, which is an advantage [22]. Because of this approach, the number of false-positives is higher and the positive predictive value (PPV) lower, even after the improvement, than approaches in other countries detecting only thyroidal CH. Dutch researchers therefore aim to improve the CH screening algorithm further by using machine learning as a tool to reduce false positives and related costs [23,24].

The addition of the second tier in the CAH algorithm prevented second heel punctures and reduced false-positive results, since the marker used in the second tier (21-DOCL) is more specific than the marker used in the first tier (17-OHP). The smaller reduction in costs in the case of CAH was caused by the relatively high costs of the second-tier testing, which are higher than the costs of a second heel puncture. Despite the smaller reduction in costs compared to the other two cases, eliminating second heel punctures for CAH is of value as it decreases the workload for screeners and medical advisors, avoids stress and anxiety in parents, and reduces unnecessary healthcare use. This shows the impact of reducing inconclusive results on other types of values besides financial benefits.

In this study, we focused on the financial consequences and did not quantify the psychosocial impact of an inconclusive and false-positive result on parents and their child. According to previous studies, parents experience more negative emotions and parental stress in the first year after a false-positive NBS result [3,4,5,6,7,8]. Furthermore, a false-positive result is linked to higher self-reported healthcare utilization until 4 months after the initial NBS result [3]. Therefore, we expect that the reduction in the number of inconclusive and/or false-positive NBS results for CH, CAH and MSUD will not only result in financial savings but will have additional value by improving health outcomes and reducing the psychosocial impact on parents and children.

Besides the financial impact and psychosocial impact, a reduction in false positives also prevents an unnecessary burden on the pediatric and laboratory healthcare system, as shown in the care pathway of a false-positive result for CH, CAH and MSUD identified by the multidisciplinary team. Preventing this avoidable healthcare utilization is particularly important given the current shortage of healthcare professionals, the aging of the population and the rising healthcare expenditures in the Netherlands [25,26]. This aligns with the goals of the Dutch Health Council to ensure appropriate care is provided in the right place and to decrease the amount of unnecessary care [26,27]. 

The numbers used for calculations in this study were based on the numbers of the evaluations published by Stroek et al., where the improved algorithms were tested in a retrospective cohort over a longer time horizon [13,14,15]. We did not use the numbers after actual implementation, because the cohort size was rather small as the implementations were very recent. However, we do not think that this will impact the results considerably, because the numbers in the evaluations of Stroek et al. were based on empirical data rather than estimates [13,14,15].

## 5. Conclusions

We conclude that recently implemented improvements in the Dutch NBS for CH, CAH and MSUD led to a substantial reduction in costs of EUR 49,832 annually. Combined with the known psychosocial effects associated with an unclear NBS finding, these results highlight the importance of evaluating screening programs and investigating possible improvements in neonatal screening algorithms.

## Figures and Tables

**Table 1:** Type of costs.

Type of Costs	Costs	Source
Medical advisor	EUR 150	Tariff From RIVM
Visit to GP	EUR 29	Reference price [17]
Visit to pediatrician and diagnostic tests to confirm CH	EUR 1024	Dutch DBC tariff [18]
Visit to pediatric endocrinologist and diagnostic tests to confirm CAH	EUR 1049	Dutch DBC tariff [18]
Visit to pediatrician for metabolic diseases and diagnostic tests to confirm MSUD	EUR 2001	Dutch DBC tariff [18]
Screener	EUR 26	Tariff from RIVM
Set for DBS	EUR 3	Tariff from RIVM
Analysis by laboratory	EUR 94	Tariff from RIVM
Second-tier analysis	EUR 290	Tariff from RIVM

GP General Practitioner; CH Congenital Hypothyroidism; CAH Congenital Adrenal Hyperplasia; MSUD Maple Syrup Urine Disease; DBS dried blood spot.

**Table 2:** Costs of false-positive CH result.

Type of Costs	Costs
Medical advisor	EUR 150
Visit GP	EUR 29
Visit pediatrician and diagnostic test to confirm CH	EUR 1024
Total	EUR 1203

GP General Practitioner; CH Congenital Hypothyroidism.

**Table 3:** Costs of inconclusive result CAH.

Type of Costs	Costs Second Heel Puncture	Costs Second Tier
Screener	EUR 26	
Set for DBS	EUR 3	
Analysis by laboratory	EUR 94	
Medical advisor	EUR 150	
Second-tier analysis		EUR 290
Total	EUR 272	EUR 290

DBS Dried Blood Spot.

**Table 4:** Costs of false-positive result CAH.

Type of Costs	Costs
Medical advisor	EUR 150
Visit GP	EUR 29
Visit pediatric endocrinologist and diagnostic tests to confirm CAH	EUR 1049
Total	EUR 1228

GP General Practitioner; CAH Congenital Adrenal Hyperplasia.

**Table 5:** Costs of false-positive result MSUD.

Type of Costs	Costs
Medical advisor	EUR 150
Visit GP	EUR 29
Visit pediatrician for metabolic diseases and diagnostic tests to confirm MSUD	EUR 2001
Total	EUR 2180

GP General Practitioner; MSUD Maple Syrup Urine Disease.

## Data Availability

Data is contained within the article or Supplementary Materials.

## Supplementary Materials

The following supporting information can be downloaded at: https://www.mdpi.com/article/10.3390/ijns10040070/s1, Supplemental file: Dutch NBS screenings algorithms for CH, CAH and MSUD.
