# Supplementary material for: The Value of Reducing Inconclusive and False-Positive Newborn Screening Results for Congenital Hypothyroidism, Congenital Adrenal Hyperplasia and Maple Syrup Urine Disease in The Netherlands
Source: Int J Neonatal Screen. 2024 Oct 8;10(4):70. doi: 10.3390/ijns10040070 (PMC11503431; doi:10.3390/ijns10040070)
Supplement: Supplementary file 1 [file IJNS-10-00070-s001.zip › IJNS-3217649-supplementary.pdf]

Supplemental File: Dutch NBS screenings algorithms for CH, CAH and MSUD

Screenings algorithm CH since March 2021 [1]

T4 in all samples, TSH in all samples with a T4 ≤ -0.8 SD (20%) of the daily mean and TBG in all samples with a T4 ≤ -1,6 SD (5%) of the daily mean.

First heel puncture:

1. T4 ≤ -3 SD of daily mean & TBG > 105 nmol/L blood = referred
2. -3 < T4 ≤ -1.6 SD of daily mean & T4/TBG ratio ≤ 17 with TSH ≥ 22 mIU/L blood = referred
   Or
   -3 < T4 ≤ -1.6 SD of daily mean & T4/TBG ratio ≤ 17 with TSH < 22 mIU/L blood = inconclusive
3. -3 < T4 ≤ -1.6 SD of daily mean & T4/TBG ratio > 17 or T4 > -1.6 SD of daily mean with TSH ≥ 22 = referred
   Or
   -3 < T4 ≤ -1.6 SD of daily mean & T4/TBG ratio > 17 or T4 > -1.6 SD of daily mean with 8 ≤ TSH < 22 mIU/L blood = inconclusive
   Or
   -3 < T4 ≤ -1.6 SD of daily mean & T4/TBG ratio > 17 or T4 > -1.6 SD of daily mean with TSH < 8 mIU/L blood = normal

Screenings algorithm CAH since October 2021 [1]

*mature babies pregnancy duration > 36 weeks:

1. First tier: 17-hydroxyprogesterone (17-OHP) ≥ 55 nmol/L blood = referred
   Or
   First tier: 17-hydroxyprogesterone (17-OHP) ≥ 25 nmol/L blood = second tier
2. Second tier: 21-deoxycortisol (21-DOCL) ≥ 2 nmol/L blood = referred

Screening algorithm MSUD since October 2021 [1]

Valine (Val) ≥ 340 μmol/L blood
AND Phenylalanine (Phe) ≥ 340 μmol/L blood
AND Leucine (Xle)/Phenylalanine (Phe) ratio ≥ 5 = referred

1. *Afkapgrenzen en beslissingscriteria neonatale screening;* National Institute for Public Health and the Environment (RIVM): 2023.
